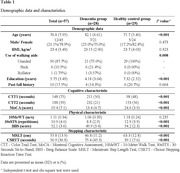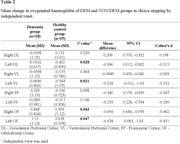# Prefrontal cortex functioning in simple and choice stepping in older adults with mild to moderate dementia – A functional near‐infrared spectroscopy (fNIRS) cross‐sectional study

**DOI:** 10.1002/alz.088611

**Published:** 2025-01-09

**Authors:** Wayne LS Chan, Michael KC Yeung, Yee Kiu Tam, Pui Man Tang, Ka Chun Wong, Ka Long Wong, Lok Yiu Wong

**Affiliations:** ^1^ The Hong Kong Polytechnic University, Hong Kong Hong Kong; ^2^ The Education University of Hong Kong, Hong Kong Hong Kong

## Abstract

**Background:**

Older adults with dementia have a high fall risk. Choice stepping (i.e., being able to execute rapid, accurate and efficient steps to reach a designated target) is an important indicator of the risk of falls in older adults. Choice stepping performance is determined by both physical (e.g., dynamic balance) and cognitive function (e.g., executive function). The prefrontal cortex may play an important role in mediating choice stepping performance. However, the prefrontal cortex activity of older adults with dementia in choice stepping is unclear.

This study aims to evaluate and compare the activity of the prefrontal cortex in simple and choice stepping in healthy older adults and older adults with mild to moderate dementia.

**Methods:**

This study was a cross‐sectional study. Twenty‐nine healthy older adults and 28 older adults with a diagnosis of mild to moderate dementia who could walk for 10 meters independently were recruited. Hemodynamic changes occurring in the participants' prefrontal cortices during simple and choice stepping were recorded by multichannel fNIRS. Choice stepping reaction time test was used to evaluate the choice stepping performance of the participants.

**Results:**

The choice stepping reaction time of the dementia group was significantly higher than that of the healthy control group (p < .001). The activations of several areas of the prefrontal cortex (left dorsolateral, left ventrolateral, left and right orbitofrontal cortices) during choice stepping tasks were significantly increased in the dementia group compared to the control group (all p < .047). A significantly higher activation in the right frontopolar cortex was also found in the choice stepping task compared to that in the simple stepping task in the dementia group (p = .040).

**Conclusion:**

Older adults with mild to moderate dementia showed a higher prefrontal cortical activation during choice stepping compared to healthy older adults. Choice stepping may demand more on the executive function of older adults with mild to moderate dementia compared to healthy older adults. Further studies using a larger sample size are required to explore the prefrontal cortical activity during different stepping tasks in older adults with dementia.